# The trimer to monomer transition of Tumor Necrosis Factor-Alpha is a dynamic process that is significantly altered by therapeutic antibodies

**DOI:** 10.1038/s41598-020-66123-5

**Published:** 2020-06-09

**Authors:** Herwin Daub, Lukas Traxler, Fjolla Ismajli, Bastian Groitl, Aymelt Itzen, Ulrich Rant

**Affiliations:** 1 Dynamic Biosensors GmbH, Lochhamer Strasse 15, 82152 Martinsried, Germany; 2 Center for Integrated Protein Science Munich, Technische Universität München, Department Chemistry, Lichtenbergstrasse 4, 85748 Garching, Germany; 3 Department of Biochemistry and Signaltransduction, University Medical Centre Hamburg-Eppendorf (UKE), Martinistrasse 52, 20246 Hamburg, Germany

**Keywords:** Biophysics, Drug discovery, Immunology, Molecular biology

## Abstract

The cytokine tumor necrosis factor-alpha (TNF-α) readily forms homotrimers at sub-nM concentrations to promote inflammation. For the treatment of inflammatory diseases with upregulated levels of TNF-α, a number of therapeutic antibodies are currently used as scavengers to reduce the active TNF-α concentration in patients. Despite their clinical success, the mode-of-action of different antibody formats with regard to a stabilization of the trimeric state is not entirely understood. Here, we use a biosensor with dynamic nanolevers to analyze the monomeric and trimeric states of TNF-α together with the binding kinetics of therapeutic biologics. The intrinsic trimer-to-monomer decay rate k = 1.7 × 10^−3^ s^−1^ could be measured directly using a microfluidic system, and antibody binding affinities were analyzed in the pM range. Trimer stabilization effects are quantified for Adalimumab, Infliximab, Etanercept, Certolizumab, Golimumab for bivalent and monovalent binding formats. Clear differences in trimer stabilization are observed, which may provide a deeper insight into the mode-of-action of TNF-α scavengers.

## Introduction

Tumor necrosis factor-alpha (TNF-α) is a cytokine that plays a key role in mediating inflammation^1–6^. Monomeric subunits of TNF-α form stable non-covalent homo-trimers at sub-nanomolar concentrations in solution^7^. Signalling proceeds by the recognition of TNF-α trimers by endogenic TNF receptors (TNFR) 1 and 2, which form trimers themselves prior to complex formation with TNF-α^8^. Trimeric TNF-α is known to monomerize over time at low concentrations, which causes a loss of bioactivity^9^. Elevated TNF-α expression levels were found in patients suffering from a multitude of different autoimmune diseases^10,11^. Scavenging antibodies and antigen-binding fragments (Fab) against TNF-α have proven to successfully suppress TNF-α-mediated inflammation in inflammatory autoimmune diseases including rheumatoid arthritis, psoriasis, and Crohn’s disease^12–16^. Despite the significant structural differences between the therapeutic antibody formats: Infliximab, Adalimumab, Certolizumab, and Etanercept, studies have shown that all four biomolecules engage in reverse signalling by binding to membrane-bound TNF-α on human cells^17^, although Etanercept and Certolizumab are unable to crosslink TNF-α trimers of any type^17–19^. Structural differences in multiple binding events of Adalimumab Fab and Infliximab Fab to a single TNF-α trimer suggest a stronger stabilization of TNF trimers in complex with Adalimumab Fab, which binds in the interface between two monomeric subunits, whereas each Infliximab Fab binds to a single monomeric subunit within a TNF-α trimer^20,21^. Cross-linking appears to be irrelevant for the induction of apoptosis as *in vitro* assays showed no significant differences between Fab and full IgG for Infliximab^22^. Assays utilizing size exclusion chromatography (SEC) could show that bivalent TNF-α binders (such as Adalimumab, Infliximab, and Golimumab) are able to form highly stable complexes of three soluble TNF-α trimers interlinked by three IgG molecules^19^. Little is known about the behaviour of TNF-α scavengers with respect to the oligomeric state of TNF-α.

In this study we investigated the dynamics of TNF-α monomerization as well as re-trimerization in real-time. Previous studies focus on monomer exchange rates in the equilibrium state, employing Förster Resonance Energy Transfer (FRET) as well as analytical size exclusion chromatographic assays^23^. In contrast to these studies, we determined a universal monomerization rate by continuous measurements in real time using a novel Electro Switchable Biosurface technique (ESB)^24^. As the monomerization of TNF-α when complexed with its scavengers reflects an increase in free TNF-α, we investigated the stability of TNF-α-trimer-TNF-scavenger complexes with Adalimumab, Infliximab, Golimumab, Certolizumab, and Etanercept as well as Fab fragments derived from Adalimumab, Infliximab and Golimumab. We find that TNF-α can quickly switch between the active trimer and inactive monomer, which reveals a new, fast-acting way to regulate its bioactivity. Interestingly, there are strong differences in the trimer stabilization of the therapeutic antibody formats and their Fabs. Some do not (fully) suppress monomerization of bound TNF-α, leading to release of TNF-α monomers. These monomeric subunits of TNF-α have not shown to form clinically relevant amounts of trimeric TNF-α. However, concentration-dependent re-trimerization has been shown *in vitro*^7^.

## Results

The complex nature of TNF-α being expressed as a trimeric membrane-bound, proteinogenic cytokine that undergoes a conversion to a soluble trimer (a strong trigger of inflammation) has been subject of several studies since the early 1990s^5–7,12,25^. These studies demonstrated a loss of bioactivity over time (monomerization of TNF-α). Additional studies could even show that bioactivity can be recovered by re-addition of TNF-α to previously monomerized TNF-α (leading to trimerization of TNF-α). However, in all studies discontinuous measurements were conducted (e.g. ELISA, analytical SEC) which do not accurately reveal the dynamics of mono- and trimerization of TNF-α on a timescale of seconds and minutes.

**Figure 1:**
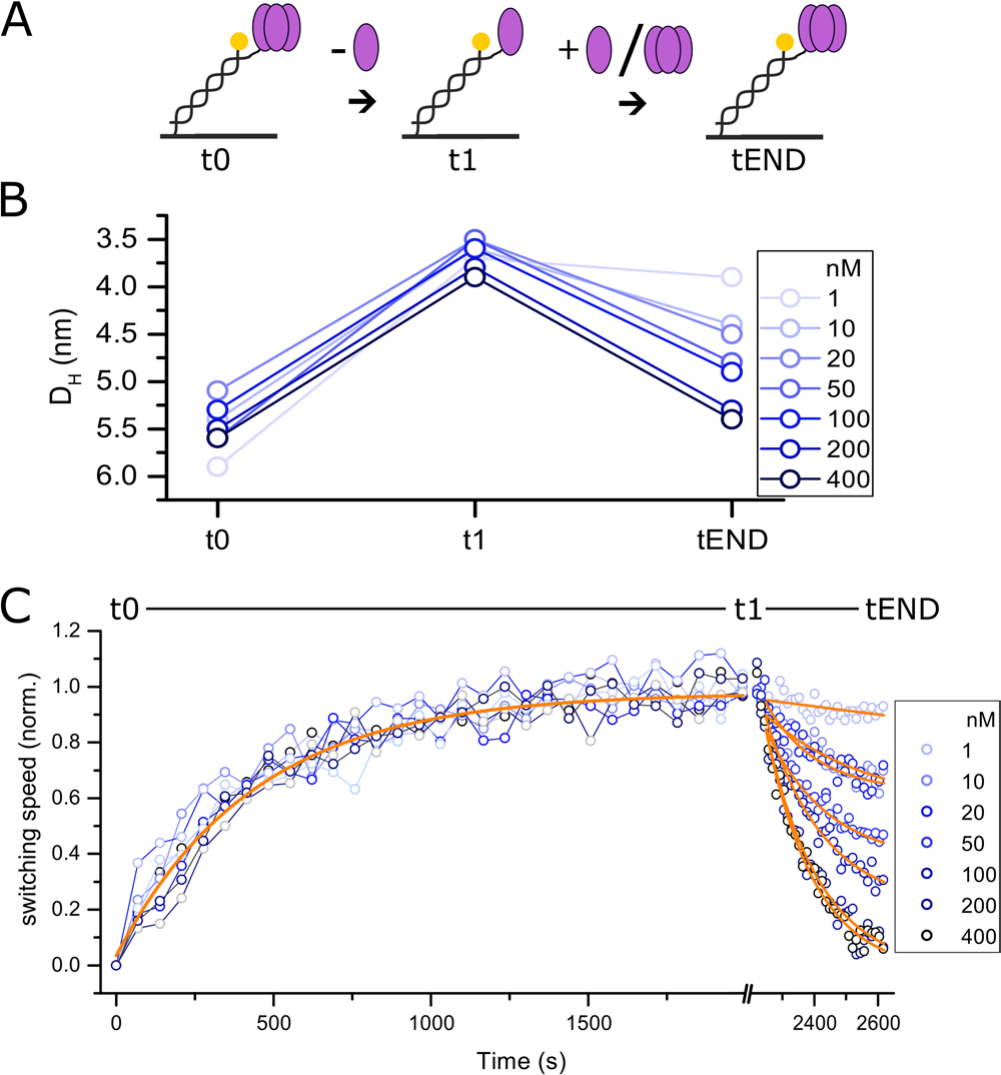
TNF-α’s monomerization and re-trimerization kinetics. TNF-α-DNA conjugates were immobilized by hybridization to surface-tethered complementary DNA strands, monomerized by buffer flow and subsequently re-trimerized (**A**). By measurement of the hydrodynamic diameter, the size of immobilized TNF-α (t0), monomerized complexes (t1), and complexes subjected to free TNF-α in nanomolar concentrations (tEND) was determined (**B**). In the initial state, size determination of immobilized TNF-α yields a hydrodynamic diameter of 5.6 ± 0.1 nm, whereas after 2000 s of buffer flow a smaller size, corresponding to monomeric TNF-α of 3.9 ± 0.1 nm is determined. Injection of higher nanomolar concentrations of free TNF-α induced efficient trimer formation, rendering the transition a reversible process. A single exponential fit of the monomerization kinetics yields a monomerization rate of 1.66 ± 0.25 E-3 s-1(C, solid orange line, t0 to t1). High nanomolar concentrations of free TNF-α led to fast re-trimerization within less than 600 s (C, t1 to tEND).

In this study, the multimerization state of TNF-α was measured using a surface biosensor with integrated microfluidic channels (Fig. 1A). Briefly, DNA nanolevers are bound to microelectrodes at one end, while TNF-α molecules are attached to their upper ends. The intrinsically charged nanolevers are dynamically actuated (switched) between lying and standing orientations by applying AC potentials to microelectrode (switchSENSE principle). Attached TNF-α molecules increase the hydrodynamic friction and hence slow the nanolever motion (switching speed), from which the hydrodynamic diameter of the bound molecules can be analyzed^26,27^.

### Monomerization of TNF-α

In order to assess the oligomeric state of TNF-α, the protein was immobilized to the sensor surface at a concentration of 500 nM, which is considerably higher than the sub-nanomolar trimerization K_D_ to avoid monomerization^7^. Before induction of monomerization, the size of immobilized TNF-α was determined in tetraplicates^26,27^, yielding a hydrodynamic diameter 5.6 ± 0.1 nm for the initial state (Fig. 1B, t0). This value is in good agreement with the computational prediction of the hydrodynamic diameter^28^ based on PDB structures of TNF-α trimers, which yield D_H_ values from 5.76 nm (3ALQ^29^) to 6.10 nm (1TNF^30^). Next, the monomerization of immobilized TNF-α was initiated by injection of running buffer for 2000 s with a flow rate of 1 ml/min, resulting in a gradual increase of the switching speed (decrease of hydrodynamic friction) due to dissociation of TNF-α subunits (Fig. 1C). Contrary to the slow monomer exchange between trimeric TNF-α in the range of hours reported previously^7,23^, the pure monomerization was found to occur within 30 minutes, yielding a monomerization rate k = 1.66 ± 0.25 10^−3^ s^−1^. After buffer flow, the monomerization efficiency was evaluated by determination of the hydrodynamic diameter of monomeric TNF-α, which yielded a D_H_ of 3.5 to 4.0 nm (t1, Fig. 1B). Again, this value was found to be in good agreement with the computational predictions based of PDB structures of monomeric TNF-α (4.39 nm, 4G3Y^21^; 4.44 nm, 3WD5^20^). This suggests that in the course of the experiment, complete monomerization was achieved.

### Re-trimerization of TNF-α

Immobilized monomeric TNF-α was subjected to different concentrations of free TNF-α to resolve the trimerization kinetics (Figs. 1C, tl-tEND). As reported earlier, the monomerization and trimerization of TNF-α is a fully reversible process^7,25^. Here, the onset of trimer formation was found to occur at approximately 10 nM free TNF-α (175 ng/ml, Fig. 1B,C). These findings are in agreement with trimer stability assessments using a FRET-based assay, showing minor loss of trimeric state at concentrations as high as 300 ng/ml under equilibrium conditions^23^. At concentrations higher than 200 nM (3.5 µg/ml) of free TNF-α, full trimer can be re-gained within minutes (Fig. 1B,C, t1-tEND).

The timescale for mono- and trimerization of TNF-α determined in these experiments is much shorter than of previously reported literature. This might reflect the normally rapidly proceeding response to pro-inflammatory triggers.

**Figure 2:**
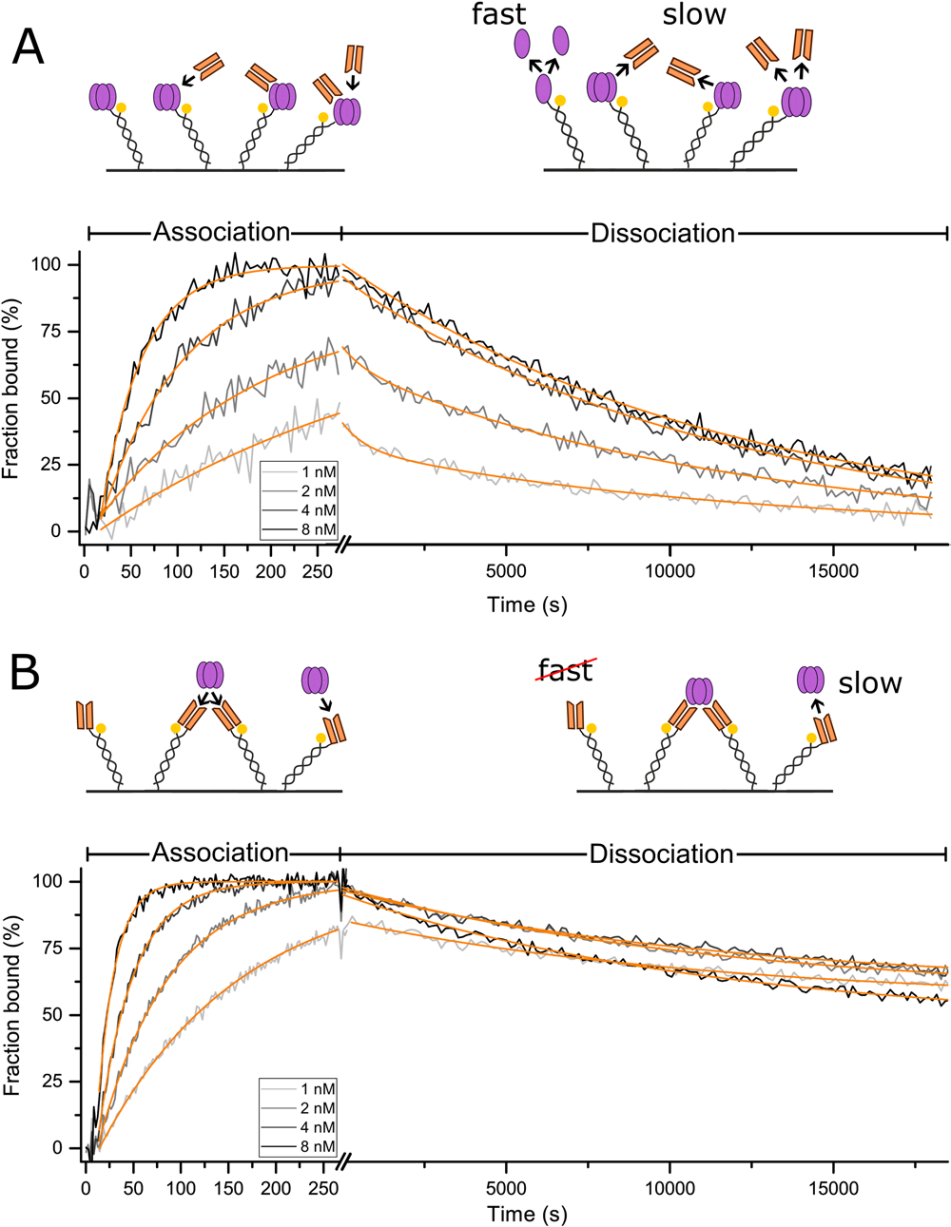
TNF-α-Adalimumab Fab interaction. Interaction of TNF-α with Adalimumab Fab was recorded in reversed (immobilized TNF-α, **A**) and in forward assay orientation (immobilized Fab fragment, **B**). Solutions of Adalimumab Fab (A) or TNF-α (B) diluted in running buffer (1, 2, 4, 8 nM) were injected for 260 s (association), followed by injection of running buffer for 18,000 s (dissociation). Solid light grey to black lines (1 to 8 nM) represent normalized data. Solid orange lines represent global fit data. Kinetic rates, affinities and amplitudes are listed in Table 1.

**Figure 3:**
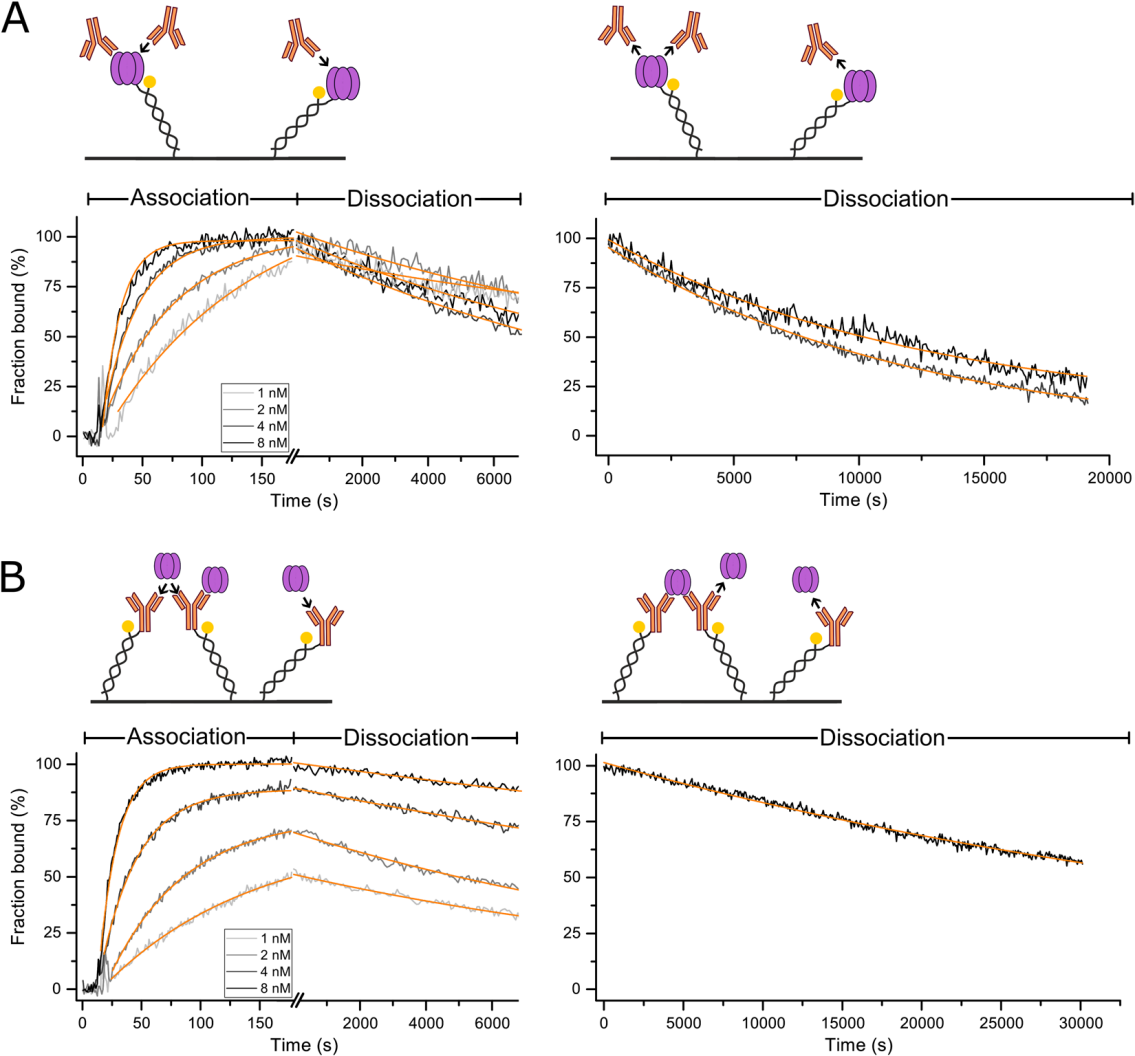
TNF-α-Adalimumab interaction. Interaction of TNF-α with Adalimumab was recorded in reversed (immobilized TNF-α, **A**) and in forward assay orientation (immobilized Adalimumab IgG, **B**). Association was recorded for 160 s, dissociation for 7200 s. Additionally, a longer dissociation phase of 18,000 s (A) and 30,000 s (B) was recorded for the highest analyte concentration. Analyte solutions were injected at concentrations of 1, 2, 4, 8 nM (light grey to black lines). Orange lines represent fit data. Kinetic rates and amplitudes are listed in Table 1.

After analysis of the dynamic nature of TNF-α’s monomerization, we investigated the impact of the oligomeric state of TNF-α on the kinetics of the most common therapeutic antibody against TNF-α, i.e. Adalimumab. To reduce the complexity of this interaction, we prepared Fab fragments of Adalimumab by limited proteolytic cleavage with Papain to create a simplified setting in which at least one of the interaction partners (here Adalimumab Fab) is monovalent. For the sake of assay simplicity, avidity (and cross-linking) effects are often avoided in literature by immobilization of the antibody^31,32^. However, for this biological system, multivalent binding cannot be ignored, but it is essential to resolve it (see cartoons, Figs. 2 and 3).

**Table 1:** Kinetic rate constants, dissociation constants.

Immobilized Ligand	Analyte in Solution	k_ON_ (10^6^ M^−1^s^−1^)	k_OFF_ (10^−5^ s^−1^)	K_D_ (pM)
TNF-α	Adalimumab Fab	2.82 (0.10)	8.52 (0.28)	30.2 (1.5)
Adalimumab Fab	TNF-α	6.73 (0.03)	8.62 (0.23)	12.8 (0.3)
TNF-α	Adalimumab	8.22 (0.15)	7.96 (0.24)	9.7 (0.3)
Adalimumab	TNF-α	7.86 (0.07)	4.60 (1.19)^a^	5.9 (1.5)

^a^Error is standard error of mean of individually fitted k_OFF_.

A comparison to literature values is drawn in the supplementary section (Table S1).

### TNF-α-Adalimumab Fab interaction

First, we investigated the simpler case of monovalent Adalimumab Fab binding to trimeric TNF-α in both orientations. In the reversed assay orientation, the association kinetics of solute Adalimumab Fab to immobilized trimeric TNF-α (Fig. 2A) could be described by a single exponential function. Each binding event to an immobilized ligand is detected independently, which corresponds to a 1:1 interaction. Dissociation kinetics of Fabs from partially saturated surfaces (injections of 1 and 2 nM Fab) are biphasic, featuring a fast and a slow rate, which are k_FAST_ = 1.42 ± 0.39 10^−3^ s^−1^ and k_SLOW_ = 8.52 ± 0.28 10^−5^ s^−1^, respectively (Figs. 2A and S3). The fast dissociation rate resembles the TNF-α monomerization rate determined before, and most likely originates from the decay of TNF-α trimers that are not stabilized by a bound Fab. The slow rate can be assigned to the dissociation of Fab from immobilized TNF-α trimers, which can be seen by comparison with the reversed the assay orientation in Fig. 2B. Here, the same rate is found for the dissociation of TNF-α from immobilized Adalimumab Fab. A fast monomerization was not observed, as expected. Rather, the observable kinetics suggest a third, even slower dissociation process, which is perceptible because the signal does not approach the baseline within the run time of 18,000 s. It could be explained by an avidity effect, where two or three immobilized Adalimumab Fabs become interlinked by one tightly bound trimeric TNF-α. It is known that multivalent binding of immobilized Adalimumab Fab forms highly stable complexes.

### TNF-α-Adalimumab interaction

We then investigated the impact of the avidity of full Adalimumab IgGs on the interaction with TNF-α. Previous studies have described the formation of highly stable complexes of three Adalimumab molecules binding to three TNF-α trimers^19^. However, the impact of these complexes on interaction parameters and whether these complexes are formed in case of one of the molecules being immobilized is subject of investigation here. In both assay orientations multiple binding can occur due to the intrinsic multivalent properties of TNF-α and Adalimumab IgGs (Fig. 3A,B). Interaction measurements of Adalimumab full IgG and TNF-α reveal comparable rate constants in both directions (Table 1). Moreover, similar to the avidity-compatible assay setup in Fig. 2B above, both orientations with Adalimumab exhibit reduced dissociation amplitudes, indicating the formation of interlinked complexes.

### Kinetic rate constants and affinities

Table 1 lists the determined kinetic rates and affinities of the interaction between TNF-α and Adalimumab Fab/full Adalimumab IgG. The dissociation rate constants are in good agreement with published data as measured for immobilized Adalimumab IgG and TNF-α in solution^19,33^. As an important result, we observed consistent affinities and similar rates for both assay orientations, showing for the first time that the orientation does not significantly influence the binding mode and its affinity.

The difference in the association rate constants of full Adalimumab IgG versus its Fab for binding to immobilized TNF-α can be explained by absence of the ‘anchor effect’ of IgG’s second binding events in case of Fab binding. Naturally, the second binding event of IgGs occurs at a drastically increased rate due to a strong increase in local concentration of the second Fab-arm upon binding of the first Fab-arm (‘forced proximity’ of second Fab)^34^.

### Adalimumab is a picomolar binder of TNF-α

**Figure 4:**
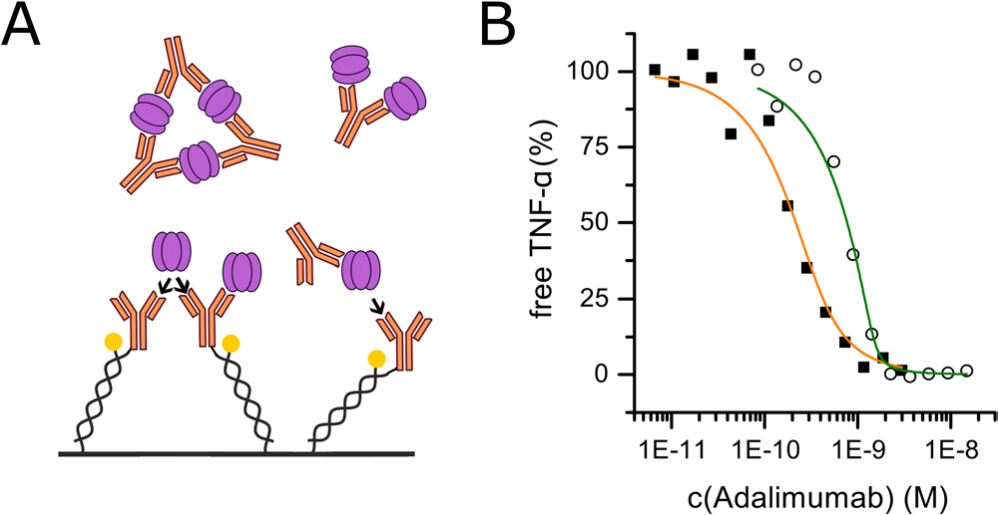
Solution equilibrium titration affinity measurement. Binding of soluble TNF-α to soluble Adalimumab (**A**). A constant concentration of TNF-α (100 pM, solid squares and 500 pM, open circles) was mixed with varying concentrations of Adalimumab (7 pM to 15 nM) and incubated for 7.5 hours. Mixtures were injected to surface-immobilized Adalimumab to determine the residual concentration of free TNF-α. Slopes of linear binding curves were normalized and plotted against soluble Adalimumab concentration. From the sigmoidal transition the dissociation constant of Adalimumab-TNF-α binding in solution is determined (K_D_(c(TNF-α) = 100 pM) = 53.6 ± 29.5 pM; K_D_(c(TNF-α) = 500 pM) <30.0 pM) (**B**).

As a complementary approach to the binding kinetics measurements described before we performed affinity measurements by solution titrations, which exclude the potential occurrence of artefacts due to the immobilization procedure^31,35^. TNF-α and antibody are mixed at different ratios in solution and allowed to bind. After equilibration, the remaining fraction of unbound TNF-α is measured by the biosensor in a concentration measurement mode. Here, we used immobilized Adalimumab to detect the remaining binding-competent TNF-α (-complexes) in pre-incubated solutions with constant concentrations of TNF-α and increasing concentrations of Adalimumab (Fig. 4A). The difficulty in performing such experiments lies in the multivalence of both binders (TNF-α trimers = 3, Adalimumab = 2), which leads to the formation of complexes of higher order, as has been described before (formation of three TNF-α trimers interlinked by three Adalimumab IgGs)^19^. Several different complexes consisting of multiple TNF-α and multiple Adalimumab molecules can be bound by surface-tethered Adalimumab, which is why the simple 1:1 fit model employed in Fig. 4A underestimates the steepness of the dose response and can only be considered an approximation. In addition, the concentration of TNF-α should be in a biologically relevant range (trimers have to be present), but low enough to still reach the K_D_-sensitive area. For the experiments here, c(TNF-α) was chosen as 100 pM and 500 pM. The solution affinity measurements confirm the dissociation constants determined in the kinetics experiments, with K_D(c(TNF-α)=100pM)_ = 53.6 ± 29.5 pM; K_D(c(TNF-α)=500pM)_ < 30.0 pM) (Fig. 4B).

### Stabilization of the TNF-α trimer by therapeutic antibody formats

**Figure 5:**
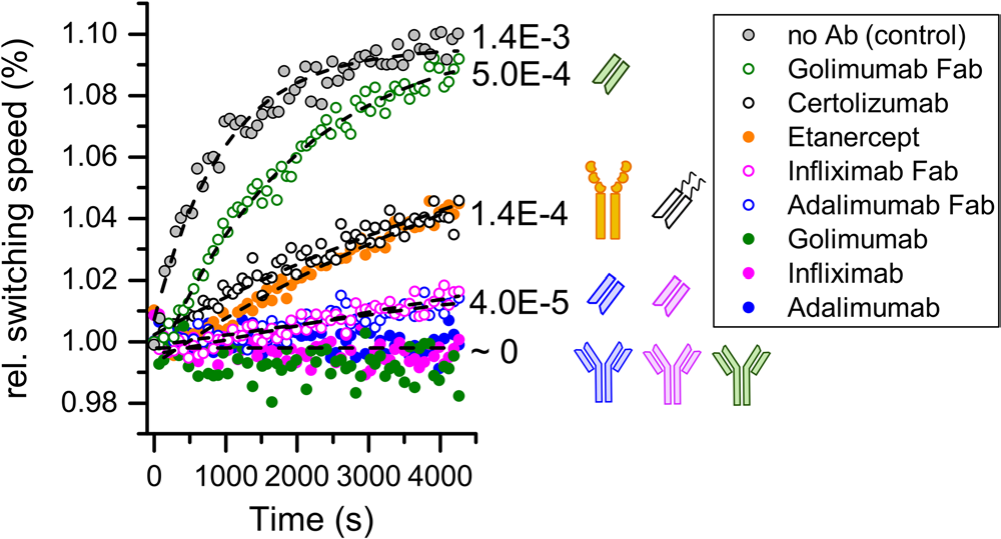
Stabilization of the TNF-α trimer by of therapeutic antibody formats. TNF-α was immobilized on the sensor surface and saturated with 5 nM of respective TNF-α-scavenger; buffer injection as no antibody control. Monomerization of TNF-α out of TNF-α-TNF-α-scavenger complexes was recorded at a constant background concentration of 5 nM of TNF- scavenger for 4200 s at a flow rate of 1 ml/min. Measurements were performed in triplicates and averaged. Monomerization rates (s^−1^) were taken from single-exponential fits (black dashed lines).

After characterizing the effects of multivalency on the interaction parameters in TNF-α-Adalimumab/Adalimumab Fab interaction, we investigated the monomerization of TNF-α in complex with each of the available five different anti-TNF-α antibodies and their Fab fragments. For this purpose, TNF-α was immobilized on the sensor surface and saturated with 5 nM of respective TNF-α scavenger. Then, the surface was continuously exposed to TNF-α scavenger (5 nM) to ensure that scavenger cannot effectively dissociate from the surface. This way, an increase in the switching speed signal indicates the decay of scavenger-bound TNF-α, but not the scavenger dissociation. The switching speed was monitored for 70 min. Full suppression of monomerization (no increase in switching speed) was observed for all avid binders of TNF-α (Adalimumab, Infliximab, Golimumab), which can form interlinked, multiple bound complexes (Fig. 5, solid blue, magenta and green symbols). These findings for Adalimumab and Infliximab are in line with literature^23^. However, in FRET assays a faster TNF-α monomerization in complex with Golimumab was found. This behaviour we only observed for the Fab fragment derived from Golimumab, which showed the weakest suppression of monomerization (Fig. 5, open green symbols). Compared to the free monomerization rate (Fig. 5, grey symbols), the rate in complex with Golimumab Fab is only reduced by a factor of three. In contrast, Fab fragments derived from Adalimumab and Infliximab showed much stronger inhibition of monomerization (Fig. 5, open blue and magenta symbols). The controlled digestion suppresses interlinking, still the monomerization rate is only just elevated to detectable levels for Infliximab Fab and Adalimumab Fab (k_MONO_ = 4.0E-5 s^−1^) (Fig. 5, open blue and magenta symbols). Moderately fast monomerization in presence of Certolizumab was also described by Van Schie^23^. Scallon *et al*. found a fast replacement of radiolabelled TNF-α in presence of Etanercept^18^. Here, both therapeutic formats which do not represent a full IgG, Certolizumab and Etanercept, showed moderately fast monomerization (Fig. 5, open black and solid orange symbols, respectively).

## Discussion

In this study, we investigated the dynamics of switching TNF-α’s oligomeric state. Based on previous studies, monomerization of TNF-α is reported to be a process happening on a time scale of hours. Although the pure monomerization rate has not been determined, merely the monitoring of the exchange of monomeric subunits between TNF-α trimers was investigated. Here, we provide data on the comparably fast transition between bioactive and -inactive forms of TNF-α on a timescale of several minutes.

This real-time assessment of monomerization may help to gain to a deeper understanding of the overall regulation of inflammation by modulation of TNF-α’s bioactivity. The highly dynamic nature of concentration-dependent adjustment of TNF-α’s oligomeric state allows for a strong immune response in defined compartments without running into the risk of a systemic inflammation. TNF-α trimers feature a built-in protective switch by rapid loss of bioactivity represented by a fast disassembly at lower serum levels. To date, an accurate determination of cytokine concentrations in biological samples remains a challenging task^36^. Thus, a comparison of absolute TNF-α concentrations of *in vitro* re-trimerization experiments shown here cannot reliably be drawn. Moreover, the rapid formation and decay of TNF-α trimers makes the accurate quantification in complex solutions even more difficult.

The general suppression of monomerization in TNF-α-Adalimumab and TNF-α-Adalimumab Fab complexes under equilibrium conditions has been characterized using HP-SEC and FRET assays^23^. However, FRET measurements cannot distinguish whether the suppression of monomerization is a product of formation of higher oligomeric complexes (e.g. thermally most stable equimolar complexes of three TNF-α trimers and three Adalimumab molecules) or caused by binding of single Adalimumab (Fab) molecules in interfaces of TNF-α monomer subunits within a trimer^19^. From kinetic experiments (Figs. 2 and 3) it is evident that the suppression of monomerization occurs in both assay orientations with both, full Adalimumab IgG and Adalimumab Fab, whenever TNF-α was saturated with the respective antagonist.

However, we observed two substantial differences between the forward and reversed assay orientation in TNF-α-Adalimumab/Adalimumab Fab interaction. Adalimumab Fab binding to immobilized TNF-α occurred at a significantly slower association rate than all the other interactions. The difference in the association rate constants of full Adalimumab IgG versus its Fab for binding to immobilized TNF-α can be described by absence of the ‘anchor effect’ of IgG’s second binding events in case of Fab binding. Naturally, the second binding event of IgGs occurs at a drastically increased rate due to a strong increase in local concentration of the second Fab upon binding of the first Fab (‘forced proximity’ of second Fab)^34^.

Moreover, there are differences in the relative dissociation amplitudes: Only if ‘bridging’ of immobilized ligand molecules can be excluded (Fig. 2A), full dissociation can be achieved.

The kinetic analyses and comparisons of Adalimumab full IgG and Adalimumab Fab interaction with TNF-α are – to our knowledge – the first interaction analyses of picomolar binders with consistent rate constants independent of assay orientation. In addition to that, full dissociation has not been shown for antibodies or Fabs with residence times >10,000 s before. Only the concerted employment of a two-dimensional ligand distribution, low surface density of immobilized antigen, and very high flow rates allowed for the suppression of otherwise inevitable re-binding of analyte, compromising both dissociation amplitude and rate. However, even by optimizing the experimental design, the characterization and quantitative analysis of two multivalent binders such as TNF-α and its scavenger antibodies remains a challenging task.

In assays with monovalent Adalimumab Fab as the soluble analyte molecule, the formation of complexes comprising multiple TNF-α trimers can be excluded. Thus, we conclude that suppression of monomerization is not just a product of the formation of complexes of high order. Our data show that the mere binding of a TNF-α scavenger to the interfacial grooves between individual units within a TNF-α trimer is sufficient to suppress monomerization.

Based on the crystal structures of Adalimumab Fab and Infliximab Fab in complex with TNF-α, a strong difference in suppression of monomerization is to be expected. Adalimumab Fab clearly binds in the interface of subunits, whereas Infliximab Fab only binds to a single subunit^20,21^. The functional analysis of trimer stabilization within this study cannot detect a difference between Adalimumab Fab or Infliximab Fab, nor between the parental IgGs (Fig. 5).

Ono *et al*. showed data that binding to the interface of monomeric subunits of TNF-α trimers is a common property of Golimumab, TNFR2 and Certolizumab^37^. Here, we observed a weaker suppression of monomerization in complexes with Golimumab Fab, Certolizumab and Etanercept. The differential stabilization of TNF-α trimers by TNF-α antagonists might play a role in the optimization of therapeutic application of these scavengers.

## Methods

### Instrumentation

All interaction measurements and size determination measurements were performed on a DRX (yellow) analyzer (Dynamic Biosensors GmbH, Martinsried, DE) at 25 °C.

### Reagents

Recombinant human TNF-α was obtained from Life Technologies (Carlsbad, CA, USA). TNF-α was supplied as lyophilized powder and reconstituted as recommended by the supplier. All therapeutic antibody formats were bought from the original provider. All protein concentrations were determined by absorbance measurements at 280 nm using a NanoDrop 2000c spectrophotometer (Thermo Fisher Scientific, Waltham, MA, USA). All solutions for interaction measurements (passivation solution, regeneration solution, buffer solutions) were provided by Dynamic Biosensors GmbH.

### Generation of fab fragments

For generation of Fab fragments, 2 mg of each respective antibody (Adalimumab, Infliximab or Golimumab) were digested using the Pierce Fab Preparation Kit (Thermo Fisher Scientific, Waltham, MA, USA), following the standard protocol. Fab fragments were reconstituted in PE140 buffer (10 mM Na_2_HPO_4_/NaH_2_PO_4_ pH 7.4, 140 mM NaCl, 50 µM EGTA, 50 µM EDTA, 0.05% (v/v) polysorbate-20) after purification.

### Protein-DNA conjugation and immobilization

Prior to the immobilization of proteins (TNF-α, Adalimumab, Adalimumab Fab) on biochip surfaces, proteins were covalently attached to 96-meric DNA oligomers (cNL-B96-01, Dynamic Biosensors GmbH) using amine coupling. Amine coupling was performed following the standard protocol (Dynamic Biosensors GmbH), whereas TNF-α and Adalimumab Fab amounts were kept at the highest recommended amount for coupling reactions (500 µg per reaction) and for Adalimumab conjugations twice the maximum amount was used (1 mg per reaction). During conjugation of TNF-α to DNA oligomers, the TNF-α-concentration was kept above 500 nM at all times, to prevent any concentration-dependent monomerization. Chromatograms of purification, following the standard protocol are shown in Figure S1. All protein-DNA conjugate concentrations were determined by absorbance measurements at 260 nm, using a NanoDrop 2000c spectrophotometer (Thermo Fisher Scientific, Waltham, MA, USA). Protein-DNA conjugates were immobilized on standard-grade biochips with 96-meric DNA anchors (MPC-96-2-Y1-S), carrying a yellow rhodamine-derivative.

### Monomerization and kinetic experiments

To minimize mass transport limitation, the probability of re-binding, and molecular crowding effects, surface densities were kept low with fluorescence amplitudes between 80–150 kcps. In addition to the reduction of the surface density, all measurements were performed at a high flow rate of 1000 µL/min to further decrease mass transport limitation and re-binding. Size determination as well as TNF-α monomerization experiments were performed in PE40 buffer (10 mM Na_2_HPO_4_/NaH_2_PO_4_ pH 7.4, 40 mM NaCl, 50 µM EGTA, 50 µM EDTA, 0.05% (v/v) polysorbate-20) and in the molecular dynamics measurement mode (alternating voltage, f = 10 kHz, calibration-corrected, V_att_ = +0.4 V; V_rep_ = −0.4 V).

Kinetic experiments of TNF-α scavengers were performed in PE140. To correct for TNF-α monomerization, interaction measurements of immobilized TNF-α with Adalimumab Fab were performed in the molecular dynamics measurement mode (alternating voltage, f = 10 kHz, V_att_ = +0.5 V; V_rep_ = −0.3 V), whereas all other interaction measurements were performed in the fluorescence proximity measurement mode (constant voltage, V_att_ = V_rep_ = −0.1 V).

### Molecular dynamics measurement mode

For determination of hydrodynamic diameters of surface-tethered proteins and protein complexes, alternating potentials are applied to induce a switching motion of DNA nanolevers (for clarification, see Figure S2). The switching motion is resolved by time-correlated single photon counting^27^. Beside the resulting fluorescence transition curves of upward and downward motion of DNA nanolevers (describing the phase of attraction of levers towards a positively charged gold electrode and repulsion from a negatively charged gold electrode), it is more practical to define a switching speed parameter (also referred to as Dynamic Response (DR)^27^).

1 $$D{R}_{t1}^{t2}={\int }_{t1}^{t2}{F}_{norm}dt$$

### In-solution equilibrium titration experiments

For each in-solution equilibrium titration experiment, the concentration of TNF-α was kept constant (100 pM and 500 pM, respectively), whereas the concentration of adalimumab was titrated from 3 pM to 8 nM. All samples were incubated for 7.5 hours to reach binding equilibrium. Unbound TNF-α was determined by injection of samples at 20 µL/min for 10 minutes in the fluorescence proximity measurement mode (constant voltage, V_att_ = V_rep_ = −0.1 V). After each injection, formed complexes were removed by application of regeneration solution (pH 13), followed by hybridization of fresh adalimumab-DNA conjugate.

### Size determination of monomerizing TNF-α

Upward motion of DNA nanolevers with and without immobilized TNF-α was recorded for 40 s with an integration time of 2 s in tetraplicates (four different detection spots within one flow channel). The hydrodynamic diameters of TNF-α were determined as described earlier^26,27^.

### Comparison of hydrodynamic diameters from ESB with structural data from the protein data bank (PDB)

The determined mean values of hydrodynamic diameters were compared to hydrodynamic diameters calculated from shell-models based on atomic coordinates^28^. Additionally, translational and rotational diffusion coefficients were calculated by Hydropro Version 10^28^, a freeware tool available here: http://leonardo.inf.um.es/macromol/programs/hydropro/hydropro.htm.

### Determination of TNF-α’s monomerization rate

Data from monomerization experiments were independently fitted using a single exponential function following Eq. 2, with k being the rate constant of monomerization.

2 $$y(t)=-A\ast \exp (\,-\,k\ast t)+y0$$

k rate constant of monomerization. A Amplitude, parameter without constraints. y0 y-Offset

### Kinetic data analysis

Recorded data traces were normalized and globally fitted by a single exponential fitting function, assuming a 1:1 binding model, unless stated otherwise. Data was processed with Origin 2015G (OriginLab Corporation, Northampton, MA, USA). k_OFF_ was determined by a global fit of the dissociation curves (Eq. 3) and subsequently used to determine k_ON_ (Eq. 4). K_D_ was calculated as k_OFF_/k_ON_.

3 $$Dissociation\,fit:y(t)=A\ast \exp (-{k}_{OFF}\ast t)+y0$$

k_OFF_ dissociation rate constant, global parameter. A Amplitude, independent parameter without constraints, if not stated otherwise. y0 y-Offset.

4 $$Association\,fit:y(t)=-\,A\ast \exp (-(c\ast {k}_{ON}+{k}_{OFF})\ast t)+y0$$

k_ON_ association rate constant, global parameter. c analyte concentration. k_OFF_ dissociation rate constant, derived from dissociation fit. A Amplitude, independent parameter without constraints, unless stated otherwise. y0 y-Offset.

### Distinction of TNF-α’s monomerization from Adalimumab Fab dissociation

The dissociation traces of the interaction of immobilized TNF-α with Adalimumab Fab were fitted with a bi-exponential function following Eq. 5, containing both the monomerization rate as well as the dissociation rate of TNF-α-monomerization (Fig. S3).

5 $$y(t)=(-{A}_{FAST}\ast \exp (-{k}_{FAST}\ast t))+(-{A}_{SLOW}\ast \exp (-{k}_{SLOW}\ast t))+y0$$

k_FAST_ rate constant of fast transition (monomerization of TNF-α), global parameter. A_FAST_ Amplitude of fast transition, parameter without constraints. k_SLOW_ rate constant of slow transition (Adalimumab Fab dissociation), global parameter. A_SLOW_ Amplitude of slow transition, parameter without constraints. y0 y-Offset.

### In-solution equilibrium titration experiments

All binding curves were normalized to their initial value and fitted with individual, linear fits. Slopes of linear binding curves were plotted semi-logarithmically against the concentration of titrated Adalimumab and subsequently fitted using Eq. 6.

6 $$y([LT])=\frac{A}{(2[RT])([RT]+[LT]+{K}_{D}-\sqrt{({([RT]+[LT]+{K}_{D})}^{2}-4[RT][LT])})}+y0$$

[RT] Total Receptor concentration; here: constant concentration of TNF-α. [LT] Total Ligand concentration; here: concentration of titrated Adalimumab. K_D_ Dissociation constant of in-solution interaction of receptor and ligand. A Amplitude. y0 y-Offset.

## Supplementary information

Supplementary Information.

## References

[1] Tracey D, Klareskog L, Sasso EH, Salfeld JG, Tak PP (2008). Tumor necrosis factor antagonist mechanisms of action: a comprehensive review. Pharmacol. Ther..

[2] Aggarwal BB (2003). Signalling pathways of the TNF superfamily: a double-edged sword. Nat. Rev. Immunol..

[3] Feldmann M, Maini RN (2002). Discovery of TNF-alpha as a therapeutic target in rheumatoid arthritis: preclinical and clinical studies. Joint, Bone, Spine Rev. Du Rhum..

[4] Locksley RM, Killeen N, Lenardo MJ (2001). The TNF and TNF receptor superfamilies: integrating mammalian biology. Cell.

[5] Saxne T, Palladino MA, Heinegãrd D, Talal N, Wollheim FA (1988). Detection of tumor necrosis factor α but not tumor necrosis factor β in rheumatoid arthritis synovial fluid and serum. Arthritis Rheum..

[6] Arend WP, Dayer JM (1990). Cytokines and cytokine inhibitors or antagonists in rheumatoid arthritis. Arthritis Rheum..

[7] Corti A, Fassina G, Marcucci F, Barbanti E, Cassani G (1992). Oligomeric tumour necrosis factor alpha slowly converts into inactive forms at bioactive levels. Biochem. J..

[8] Chan FK (2000). A domain in TNF receptors that mediates ligand-independent receptor assembly and signaling. Science.

[9] Narhi LO, Arakawa T (1987). Dissociation of recombinant tumor necrosis factor-alpha studied by gel permeation chromatography. Biochem. Biophys. Res. Commun..

[10] Van Deventer SJ (1997). Tumour necrosis factor and Crohn’s disease. Gut.

[11] Schottelius AJG (2004). Biology of tumor necrosis factor-alpha- implications for psoriasis. Exp. Dermatol..

[12] Brennan FM, Chantry D, Jackson A, Maini R, Feldmann M (1989). Inhibitory effect of TNF alpha antibodies on synovial cell interleukin-1 production in rheumatoid arthritis. Lancet (London, England).

[13] Elliott MJ (1993). Treatment of rheumatoid arthritis with chimeric monoclonal antibodies to tumor necrosis factor alpha. Arthritis Rheum..

[14] Elliott MJ (1994). Randomised double-blind comparison of chimeric monoclonal antibody to tumour necrosis factor alpha (cA2) versus placebo in rheumatoid arthritis. Lancet (London, England).

[15] Feldmann M, Maini RN (2001). Anti-TNF alpha therapy of rheumatoid arthritis: what have we learned?. Annu. Rev. Immunol..

[16] Suenaert P (2002). Anti-tumor necrosis factor treatment restores the gut barrier in Crohn’s disease. Am. J. Gastroenterol..

[17] Nesbitt A (2007). Mechanism of action of certolizumab pegol (CDP870): *in vitro* comparison with other anti-tumor necrosis factor alpha agents. Inflamm. Bowel Dis..

[18] Scallon B (2002). Binding and functional comparisons of two types of tumor necrosis factor antagonists. J. Pharmacol. Exp. Ther..

[19] Santora LC, Kaymakcalan Z, Sakorafas P, Krull IS, Grant K (2001). Characterization of noncovalent complexes of recombinant human monoclonal antibody and antigen using cation exchange, size exclusion chromatography, and BIAcore. Anal. Biochem..

[20] Hu S (2013). Comparison of the Inhibition Mechanisms of Adalimumab and Infliximab in Treating Tumor Necrosis Factor α-Associated Diseases from a Molecular View. J. Biol. Chem..

[21] Liang S (2013). Structural basis for treating tumor necrosis factor α (TNFα)-associated diseases with the therapeutic antibody infliximab. J. Biol. Chem..

[22] Lügering A (2001). Infliximab induces apoptosis in monocytes from patients with chronic active Crohn’s disease by using a caspase-dependent pathway. Gastroenterology.

[23] van Schie, K. A. *et al*. Therapeutic TNF Inhibitors can Differentially Stabilize Trimeric TNF by Inhibiting Monomer Exchange. *Sci. Rep*. **6**, (2016). 10.1038/srep32747 PMC5015024 27605058

[24] Knezevic J (2012). Quantitation of affinity, avidity, and binding kinetics of protein analytes with a dynamically switchable biosurface. J. Am. Chem. Soc..

[25] Hlodan R, Pain RH (1995). The folding and assembly pathway of tumour necrosis factor TNF alpha, a globular trimeric protein. Eur. J. Biochem..

[26] Langer A (2013). Protein analysis by time-resolved measurements with an electro-switchable DNA chip. Nat. Commun..

[27] Langer A, Kaiser W, Svejda M, Schwertler P, Rant U (2014). Molecular Dynamics of DNA-Protein Conjugates on Electrified Surfaces: Solutions to the Drift-Diffusion Equation. J. Phys. Chem. B.

[28] Ortega A, Amorós D, García de la Torre J (2011). Prediction of Hydrodynamic and Other Solution Properties of Rigid Proteins from Atomic- and Residue-Level Models. Biophys. J..

[29] Mukai, Y. *et al*. Solution of the Structure of the TNF-TNFR2 Complex. *Sci. Signal*. **3**, ra83 LP-ra83 (2010). 10.1126/scisignal.2000954 21081755

[30] Eck MJ, Sprang SR (1989). The structure of tumor necrosis factor-alpha at 2.6 A resolution. Implications for receptor binding. J. Biol. Chem..

[31] Drake AW, Myszka DG, Klakamp SL (2004). Characterizing high-affinity antigen/antibody complexes by kinetic- and equilibrium-based methods. Anal. Biochem..

[32] Drake AW (2012). Biacore surface matrix effects on the binding kinetics and affinity of an antigen/antibody complex. Anal. Biochem..

[33] Kaymakcalan Z (2009). Comparisons of affinities, avidities, and complement activation of adalimumab, infliximab, and etanercept in binding to soluble and membrane tumor necrosis factor. Clin. Immunol..

[34] Vauquelin G, Charlton SJ (2013). Exploring avidity: understanding the potential gains in functional affinity and target residence time of bivalent and heterobivalent ligands. Br J Pharmacol..

[35] Nieba L, Krebber A, Plückthun A (1996). Competition BIAcore for measuring true affinities: large differences from values determined from binding kinetics. Anal. Biochem..

[36] Aziz N (2016). Stability of cytokines, chemokines and soluble activation markers in unprocessed blood stored under different conditions. Cytokine.

[37] Ono M (2018). Structural basis for tumor necrosis factor blockade with the therapeutic antibody golimumab. Protein Sci..

