# Supplementary material for: The trimer to monomer transition of Tumor Necrosis Factor-Alpha is a dynamic process that is significantly altered by therapeutic antibodies
Source: Sci Rep. 2020 Jun 9;10:9265. doi: 10.1038/s41598-020-66123-5 (PMC7283243; doi:10.1038/s41598-020-66123-5)
Supplement: Supplementary file 1 — Supplementary Information. [file 41598_2020_66123_MOESM1_ESM.pdf]

Supplementary Information (SI)

The trimer to monomer transition of Tumor Necrosis Factor-Alpha is a dynamic process that is significantly altered by therapeutic antibodies

Herwin Daub[1,2*], Lukas Traxler[1], Fjolla Ismajli[1], Bastian Groitl[1], Aymelt Itzen[2,3], Ulrich Rant[1]

[1] Dynamic Biosensors GmbH, Lochhamer Strasse 15, 82152 Martinsried, Germany
[2] Center for Integrated Protein Science Munich, Technische Universität München, Department Chemistry, Lichtenbergstrasse 4, 85748 Garching, Germany
[3] Department of Biochemistry and Signaltransduction, University Medical Centre Hamburg-Eppendorf (UKE), Martinistrasse 52, 20246, Hamburg, Germany
[*]daub@dynamic-biosensors.com

Figure S1. Chromatograms of Ion Exchange purifications of TNF-α (**A**), Adalimumab Fab (**B**) and Adalimumab (**C**). Elution of TNF-α shows one conjugate peak, corresponding to pre-dominantly intact trimer (Fig. 1B). Also, the chromatogram of Adalimumab Fab shows a single peak (**B**). The conjugation of Adalimumab full IgG shows a minor second conjugate peak (elution volume: 9.86 ml), which was not used during measurements (**C**).

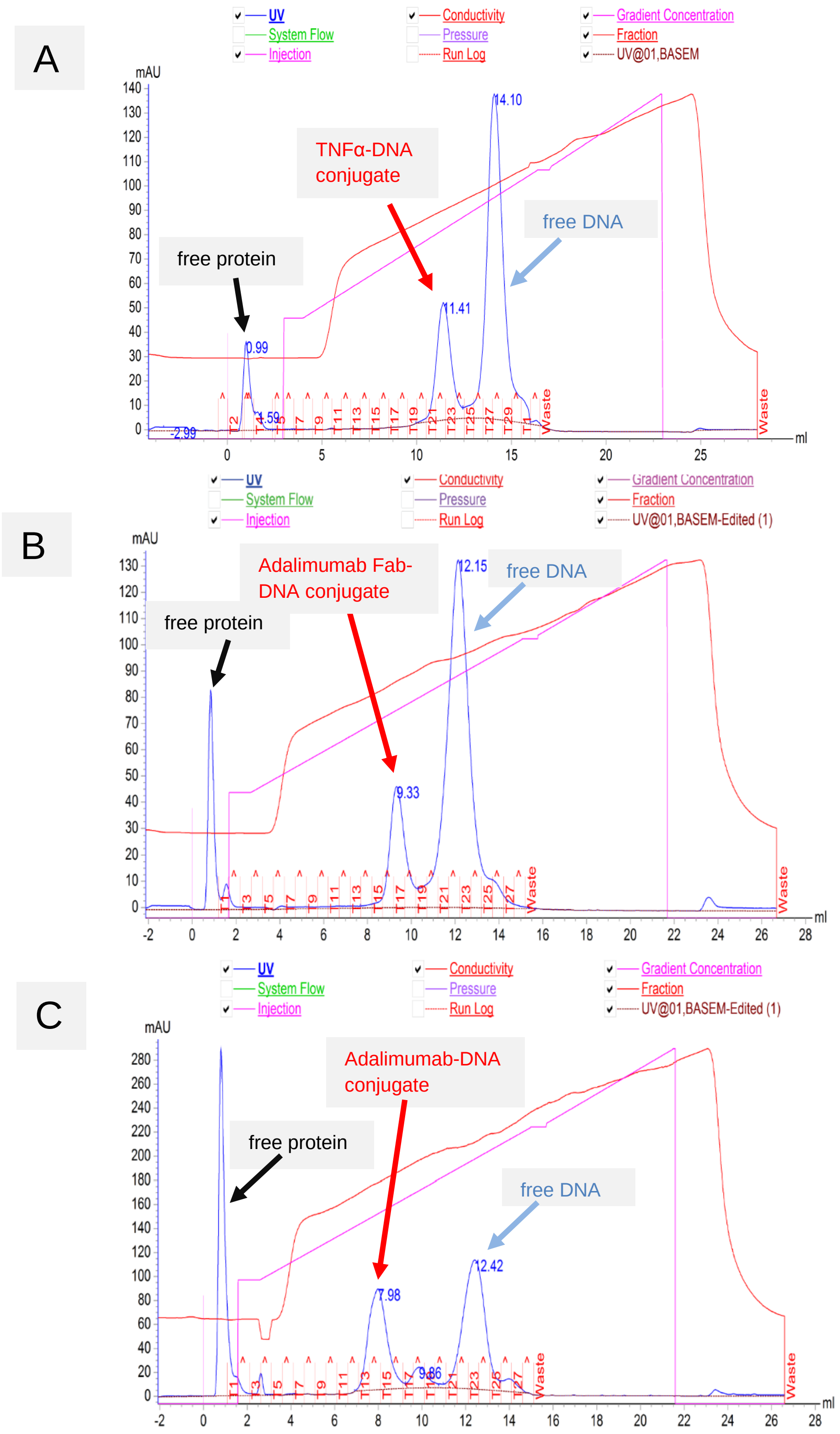

Figure S2. Switching Cycle of DNA (black line) and DNA with protein cargo (orange line). As indicated in the cartoons, the first 50 µs a positive potential of +400 mV is applied, which attracts DNA nanolevers. By approaching the gold surface, the quenching efficiency is increasing over time, thus the fluorescence emission is decreased. From 50 to 100 µs, a negative potential repels the DNA from the surface. The first part of this transition is used to define the dynamic response (see inset). As described in the materials section, the integral of normalized fluorescence (or the area below the curve) is used to define the switching speed.

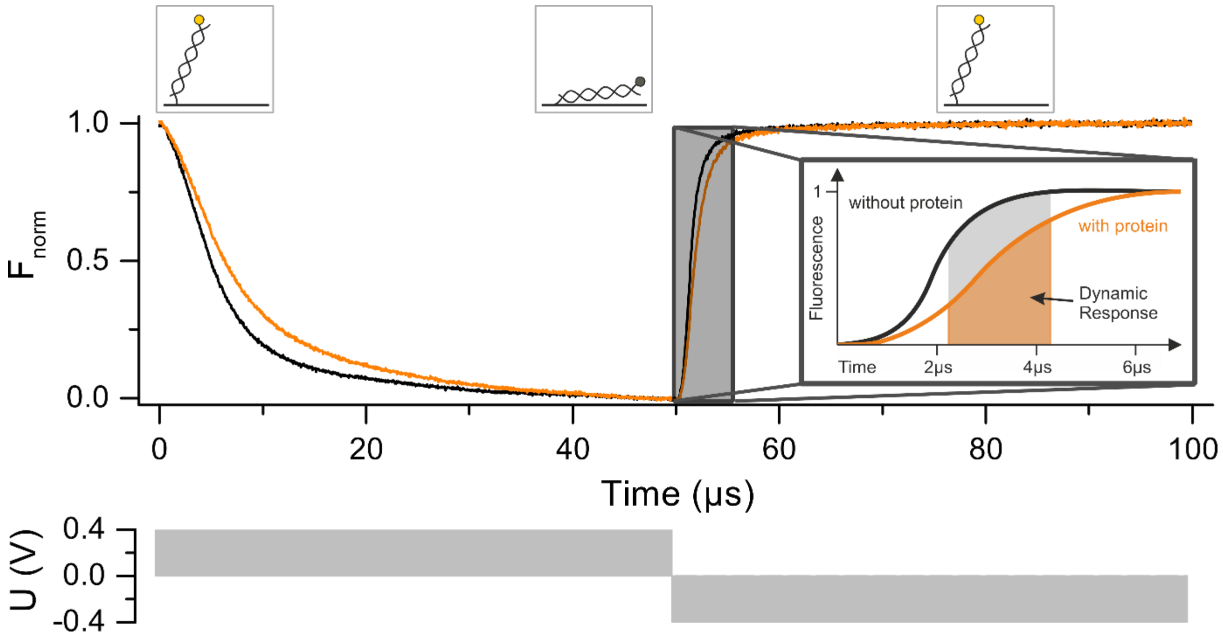

Figure S3. Dissociation sensorgram of Adalimumab Fab dissociation from immobilized TNF-α (**A**). Fully complexed TNF-α does not exhibit any monomerization due to suppression by Fab binding. Dissociation curves of fully complexed TNF-α can be fit with a single-exponential function only (black, top curve; 8 nM Adalimumab Fab injection) (**A**). Monomerization prior to Fab dissociation can be found in dissociation curves of partially saturated TNF-α. The two transitions, TNF-α monomerization (blue, dotted line) and Adalimumab Fab dissociation (red, dashed line), occur on different timescales as exemplarily displayed in the bi-phasic dissociation curve of 1 nM Adalimumab Fab from immobilized TNF-α (light grey) (**A**). Note that the monomerization does only describe the first part of the curve, whereas the latter part is dominated by dissociation of Adalimumab Fab. Strikingly, bi-exponential fitting reveals for a bigger fraction of uncomplexed TNF-α or "Fraction free", the inverse of Fraction bound, a bigger amplitude for the fast transition rate or an increasing amount of TNF-α monomerization (red arrow) (**B**). Additionally, full dissociation of Adalimumab Fab can be resolved here because no interlinking by multivalent analyte molecules is possible (**C**). This contrasts with experiments in which Adalimumab Fab was immobilized and thus interlinking is possible (Fig. 2B).

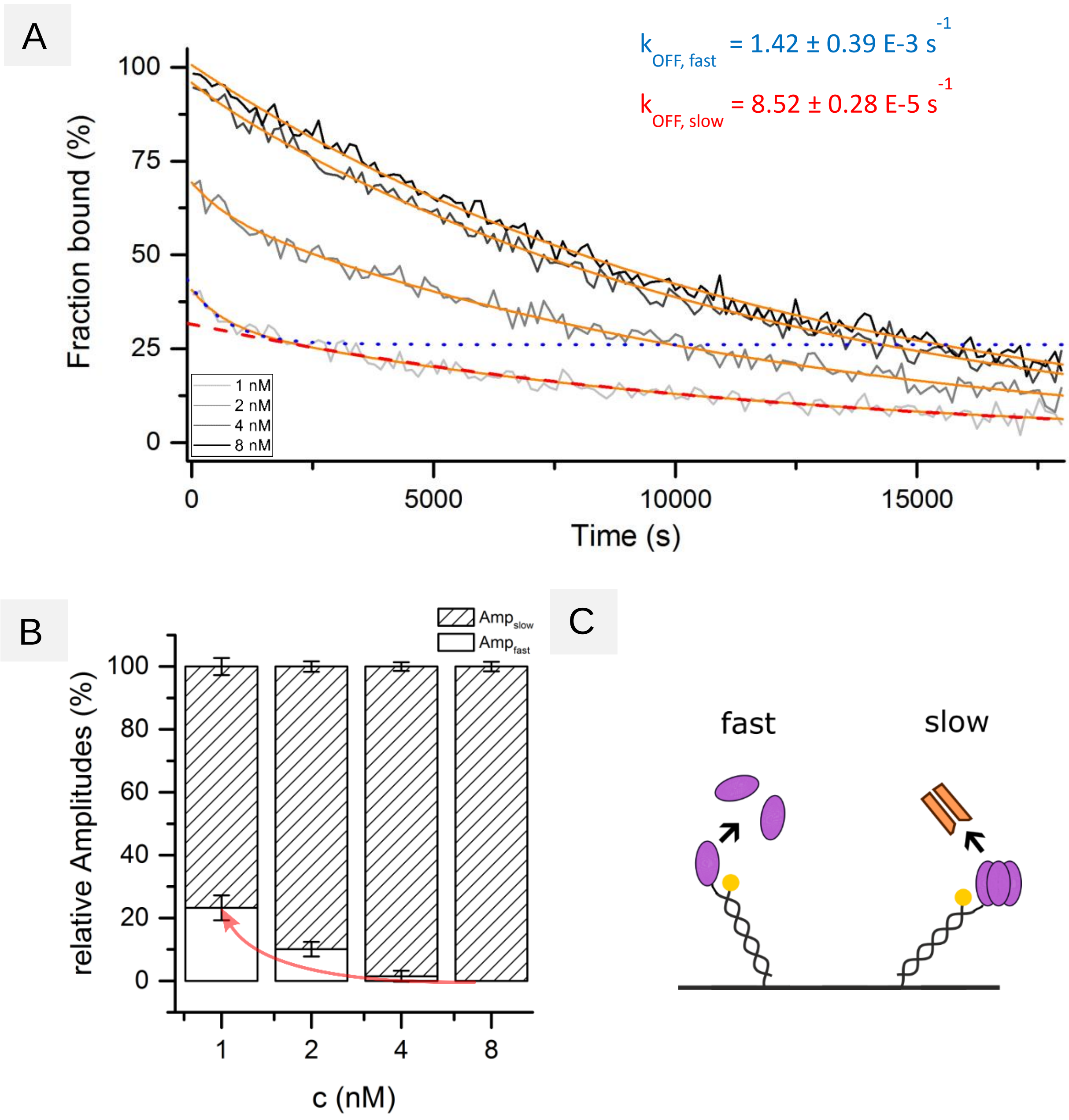

Table S1. Excerpt of published values for rate constants and dissociation constants of TNF-α and Adalimumab interaction.

Technique	Immobilized Ligand	Analyte in Solution	k_{ON} (10^6 $M^{-1}s^{-1}$)	k_{OFF} (10^{-5} s^{-1})	K_D (pM)	Reference
ESB	Adalimumab	TNF-α	7.86 (0.07)	4.60 (1.19)[b]	5.9 (1.5)	This work [Fig. 3B]
SPR	Adalimumab	TNF-α	1.69	4.71	30.4	(33)
ESB	TNF-α	Adalimumab	8.22 (0.15)	7.96 (0.24)	9.7 (0.3)	This work [Fig. 3A]
SPR	TNF-α	Adalimumab	n.d.	n.d.	n.d.	
KinExA		In-solution assay	N/A	N/A	8.6	(33)
ESB		In-solution assay	N/A	N/A	~40	This work [Fig. 4B]

For better comparison, table S1 shows an excerpt of published values for interaction parameters between TNF-α and Adalimumab. Immobilization techniques used for measurements in SPR and ESB appear to have no or only a minor effect on the dissociation constant as they compare very well to in-solution assays (e.g. KinExA). Please note, for both in-solution assays listed here, possible deviations through formation of asymmetric complexes of higher order, as mentioned in results section apply.
